# Combined use of intravenous and topical tranexamic acid in patients aged over 70 years old undergoing total hip arthroplasty

**DOI:** 10.1186/s13018-019-1384-6

**Published:** 2019-11-07

**Authors:** Junqing Jia

**Affiliations:** Department of Orthopedics, Shanxi Great hospital, Taiyuan, 030032 Shanxi Province China

**Keywords:** Tranexamic acid, Total hip arthroplasty, Blood loss, Combined treatment

## Abstract

**Purpose:**

The present study was designed to evaluate the efficacy and safety of combined use of intravenous (IV) TXA administration and topical intraarticular tranexamic acid (TXA) strategy in patients aged over 70 undergoing total hip arthroplasty (THA).

**Methods:**

One hundred eighty patients were randomized into three groups, including an IV group, a local group, and a combined group. Patients were administrated with 15 mg/kg of IV-TXA in the IV group, 2 g TXA in the topical group, or 15 mg/kg IV-TXA combined with 2 g TXA in the combined group. Total blood loss (TBL), maximum hemoglobin drop, the transfusion rate and the number of allogeneic blood units, and the incidence of deep venous thrombosis (DVT), and pulmonary embolism (PE) were recorded and analyzed.

**Results:**

TBL was 757.75 ± 188.95 mL in the combined group, which was significantly lower than in the IV group (892.75 ± 218.47) or the topical group (1015.75 ± 288.71) (*p* = 0.015, *p* = 0.001 respectively). The mean values of maximum hemoglobin drop in the combined, IV, and topical groups were 2.67 ± 0.42, 3.28 ± 0.52, and 3.75 ± 0.62 g/dL, respectively, with a significant intergroup difference (*p* < 0.001 for all). PE was not detected within 1 month after the surgery. Asymptomatic DVT was reported in 1 patient of the IV group, and in 2 patients from the combined group, while the difference was not statistically significant.

**Conclusions:**

Compared to intravenous or topical use of TXA, the combined therapy effectively decreased total blood loss and reduced the transfusion rate, simultaneously possessed the same degree of safety in primary THA patients aged over 70.

## Introduction

Total hip arthroplasty (THA) has been one of the most satisfying procedures in current orthopedic practice, especially for patients suffering from various painful hip disease, THA can relieve pain and restore the function of the broken hip, and thus improve patients’ life quality substantially [1].

However, THA is usually associated with substantial blood loss, especially in the first week after surgery, which often results in anemia [2], and the patients suffer a higher risk of cardiopulmonary events and longer recovery period, and higher health care costs [2–4]. Thus, blood transfusions are usually required, although blood transfusions are accompanied by an unwelcome immunologic reaction and infectious disease transmission [3, 5].

Various means like hypotensive anesthesia, preoperative autologous blood transfusion, and using antifibrinolytic agents had been used to reduce blood loss in THA [6]. Especially, tranexamic acid (TXA) is widely used. By blocking lysine-binding sites of plasminogen, TXA suppresses fibrinolysis, thus led to reduced proteolytic activity on the fibrin monomers and fibrinogen and reduced blood loss [7–11].

The administration strategies of TXA are in constant change and development. Traditionally, THA is administered intravenously, and the practice has been populated over time [12–16]. The intravenous (IV) administration of TXA has reduced blood transfusion rate by 38% in various orthopedic surgeries [17, 18]. THA could also decrease blood loss and blood transfusion rate without increasing the incidence of deep vein thrombosis (DVT) and pulmonary embolism (PE) [19–21]. In addition, it was suggested TXA concentrations up to 10 mg/mL of in vivo do not influence the platelet count, the coagulation time, or other coagulation factors [3].

However, several studies reported concerns of the symptomatic TE events remained unclear due to IV administration of TXA [22–26]. As aged people are more prone to develop thrombus because of increased blood viscosity, hypertension, and arteriosclerosis [27]. In this regard, the theoretical risk of thromboembolic complications does exist because most THA operations were processed on aged people [28].

Multiple studies argued that the topical use of TXA could achieve similar or even better hemostatic efficacy than intravenous use of TXA [29–33]. It is suggested that topical use TXA could directly target the bleeding sites and accumulate at the surgical area. Recently and interestingly, studies had demonstrated the combined therapy possesses the highest hemostatic efficacy [12, 34, 35]. However, we are still in short of solid scientific evidence related to the safety of the combined strategy, especially in patients over 70 years old.

All the above, we conducted the present study in patients undergoing total hip arthroplasty over 70 years old, and all the patients were randomized into three groups, including an IV group, a topical group, and a combined group. The hemostatic efficacy of the above three groups was compared and analyzed, based on total blood loss and the transfusion rate. In addition, the incidence of DVT and PE were recorded and compared to evaluate the safety of each regimen in patients over 70 years old. As the combined use of TXA-enhanced antifibrinolytic activity systematically and locally, we hypothesized that the combined regimen of intravenous and topical TXA would achieve less total blood loss, reduced blood transfusion rate, and lower hemoglobin (Hb) drop.

## Materials and methods

### Patients and grouping

**Fig. 1:**
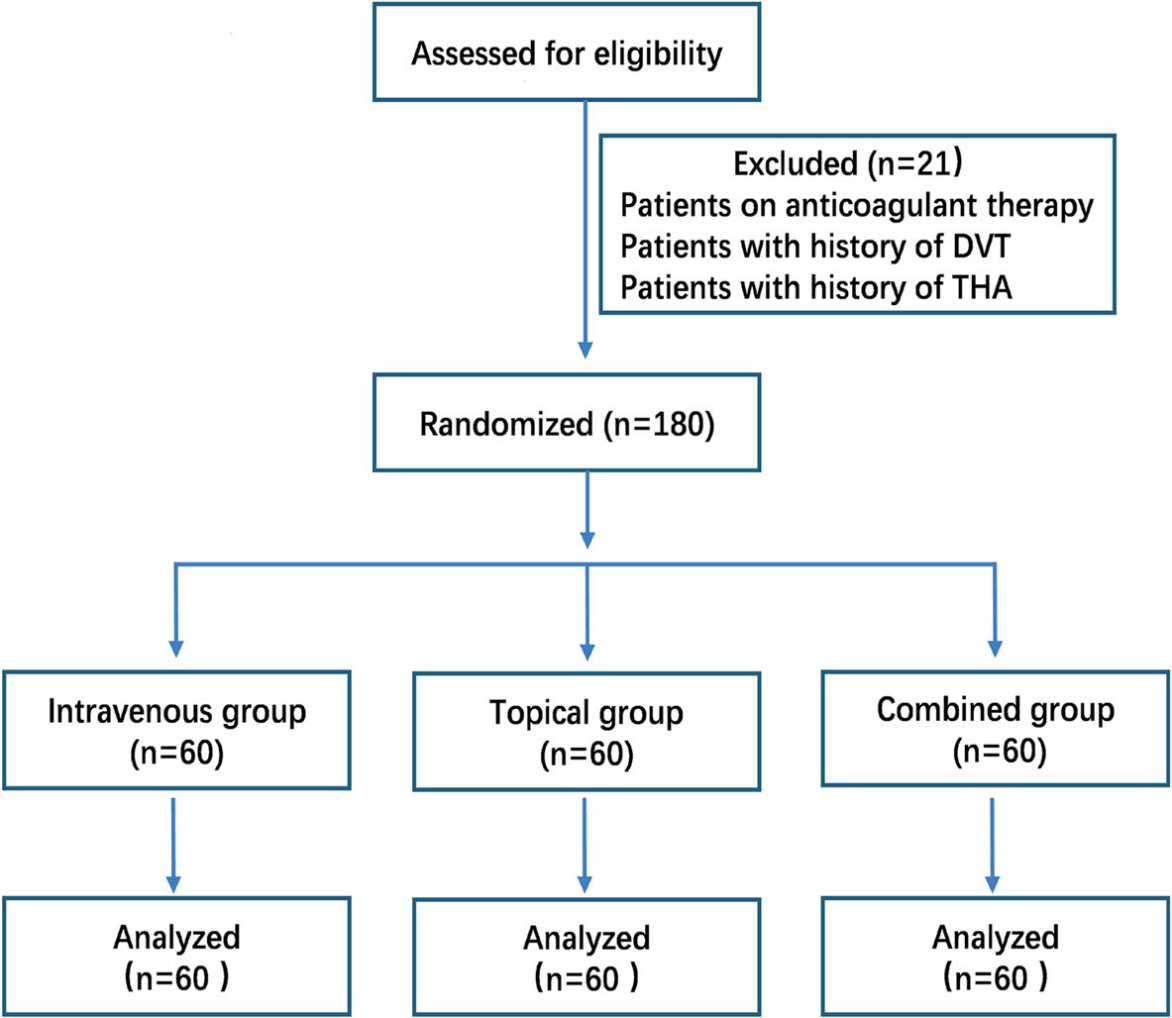
Flow of patients through the study

A total of 201 consecutive patients aged over 70 scheduled for elective unilateral primary THA were assessed for eligibility from September 2010 to December 2017 (Fig. 1). All the patients aged over 70 and diagnosed with hip osteoarthritis or femoral head osteonecrosis were included in this study, and patients were further excluded if they were diagnosed with coagulopathy; accepting ongoing anticoagulation therapy; possessing thromboembolic disease history, and complaining hepatic or renal dysfunction or ischemic heart disease history. After meeting the inclusion and exclusion criteria,180 patients were enrolled and randomized into three groups, intravenous (IV) TXA alone (group I), topical TXA (group II), and combined intravenous and intraarticular TXA (IV plus IA, group III). Thus, 60 patients were assigned to the IV group, 60 patients to the topical group, and 60 to the combined IV and topical group. The demographic statistics, preoperative hematologic data, and time of operation of the three groups were recorded and then shown in Fig. 1.

### Administration of TXA

Patients in the IV group were given a single intravenous dose of TXA at 15 mg/kg of body weight 30 min prior to skin incision. For patients assigned to the topical group, 2 g TXA dissolved in 30 mL of 0.9% sodium chloride saline was injected into the joint space 5 min after incision closure. Patients in the combined group were given a single intravenous dose of TXA and 2 g TXA in local.

### Surgical procedure and perioperative management

All surgical procedures were conducted by the same surgical team using the posterior approach. Cementless acetabular cups and femoral stems (Pinnacle cup, Corail stem; Depuy Synthes, USA) were applied, and all procedures were performed under general anesthesia by the same posterolateral approach.

The drainage of all groups remained clamped for 6 h and was removed 24 h post-surgery. Two thousand international units of low molecular weight heparin was first administrated 8 h post-surgery and repeated every 24 h with 4000 IU until discharge. All the patients were helped with an intermittent pneumatic compression pump from day 1 post-surgery. Clinical symptoms related to DVT were monitored before discharge. After discharge, the patients were prescribed with 10 mg Rivaroxaban daily for 30 days. Ultrasound was also used to detect DVT on the day of discharge, 1-, and 3-month follow-up. In addition, ultrasound was performed immediately for DVT, and computed tomography was taken to examine PE if any suspicious symptom were complained.

### Blood transfusion guidelines

Blood transfusions were performed according to a protocol based on the management guidelines for perioperative transfusion published by the Chinese Ministry of Health. Based on the guidelines, a blood transfusion was required when the hemoglobin was lower than 70 g/L, or when hemoglobin was lower than 80 g/L, but the patient developed symptomatic anemia, such as lightheadedness, presyncope, fatigue, palpitations, or shortness of breath.

### Outcome assessment

The primary outcomes measures include total blood loss (TBL), the allogeneic blood transfusion rate, maximum hemoglobin drop, hematocrit change, and the incidence of DVT and PE. Total blood loss was calculated based on patient blood volume, Hb loss, and a previously reported equation [15, 36]. The secondary outcomes included the length of hospital stay, complications (wound leakage, hematoma, superficial infection, and deep infection), and other adverse events (cardiac infarction, stroke, and acute renal failure). Ultrasonographic examination was performed if patients complain about suspicious symptoms, including pain, swelling, and tenderness in the lower limb.

### Statistical analysis

The SPSS software (version 20.0; IBM, Chicago, IL) was used to compare the statistical difference. The one-way ANOVA and Tukey’s post hoc test was used to determine the significances of group differences in continuous variables. The Pearson chi-squared test or Fisher exact test was applied to analyses qualitative comparative parameters. A significant difference was defined as *p* < 0.05.

## Results

In total, 201 patients aged over 70 years old primary were performed with THA in our hospital from September 2010 to December 2017. Twenty-one patients were excluded according to the exclusion criteria. The remaining 180 patients were included and followed up, with 60 patients randomized to the IV group, 60 to the topical group, and 60 to the combined group (Fig. 1).

Demographic characteristics regarding age, gender, body mass index (BMI), ASA grade, and preoperative laboratory parameters were compared between the three groups (Table 1). Primary outcome data and complications of all the groups were presented in Tables 2 and 3. As Table 2 showed, the combined group had a significantly lower TBL and a smaller reduction in hemoglobin concentration and hematocrit compared to the other two groups. TBL was 757.75 ± 188.95 mL in the combined group, which was significantly lower than in the IV group 892.75 ± 218.47 mL or the topical group 1015.75 ± 288.71 mL (*p* = 0.015, *p* = 0.001 respectively). In addition, the average maximum hemoglobin drop was 2.67 ± 0.42, 3.28 ± 0.52, and 3.75 ± 0.62 g/dL in the combined, IV, and topical groups respectively, with a significant intergroup difference (*p* < 0.01 for all). As for the blood transfusion rate, only one patient in the combined group required a blood transfusion. In contrast, 6 patients in the IV group, and 7 patients in the topical group required blood transfusions. No PE was detected during the 2-month follow-up, and asymptomatic DVT was reported by 1 patient of the IV group, and in 2 patients from the combined group. The intergroup difference was not statistically significant (*p* = 0.774).

**Table 1:** Baseline characteristics of the study population

	Variable	Combined Group (*N* = 60)	Single IV Group (*N* = 60)	Topical Group (*N* = 60)	*p*
Demographic characteristics	Age (years)	75.44 ± 4.75	76.34 ± 3.55	75.61 ± 5.27	0.46
	Male sex (no. [%] of patients)	34(58%)	27 (45%)	33(55%)	0.51
	Weight (kg)	62.68 ± 11.49	57.75 ± 11.32	60.38 ± 9.98	0.08
	Height (cm)	162.20 ± 8.45	158.50 ± 9.12	160.74 ± 7.84	0.06
	Body mass index (kg/m^2^)	23.70 ± 3.04	22.95 ± 3.81	22.95 ± 2.21	0.42
	Osteonecrosis of femoral head	31	35	33	–
	Osteoarthritis	29	25	27	–
Preop. laboratory values	Hemoglobin (g/dL)	130.21 ± 14.91	132.57 ± 17.23	131.49 ± 15.68	0.28
	Hematocrit (L/L)	0.45 ± 0.05	0.43 ± 0.06	0.44 ± 0.03	0.34
	Platelet count (109/L)	178.68 ± 62.43	173.76 ± 51.17	167.12 ± 66.32	0.88

*p* values indicate that were significantly different between the groups

**Table 2:** Primary outcomes regarding blood loss

Variable	Combined group (*N* = 60)	Single IV group (*N* = 60)	Topical group (*N* = 60)	*p*
hemoglobin drop	2.67 ± 0.42	3.28 ± 0.52	3.75 ± 0.62	< 0.05
Platelet count (109/L)	138.12 ± 55.78	134.24 ± 49.29	139.52 ± 50.90	0.30
Total blood loss (mL)	757.75 ± 188.95	892.75 ± 218.47	1015.75 ± 288.71	< 0.05

The values are given as the mean and standard deviation. *p* values indicate a significant difference among groups

**Table 3:** Primary outcomes regarding transfusion

Variable	Combined group (*N* = 60)	Single IV group (*N* = 60)	Topical group (*N* = 60)	*p*
No. (%) of patients given transfusion	1 (1.67%)	6 (10%)	8 (13.33%)	< 0.05
No. of units transfused	3	15.0	20	< 0.05
Amount of transfusion (mL)	600	3000	8000	< 0.05

The values are given as the mean and standard deviation. *p* values indicate a significant difference among groups

As for the secondary outcomes presented in Table 3, diverse events like cardiac infarction, stroke, or acute renal failure had not been observed during the two-month follow-up. Mild wound leakage was observed postoperatively in 13 patients in all the groups (4 patients in the combined group, 5 patients in the IV group, and 4 patients in the topical group), and the analysis of the results showed no statistical differences. All the wound leakage were controlled with wound dress changing. Other complications were not reported. All three groups presented similar results regarding hospital stay and cost, with no statistically significant differences after comparison.

## Discussion

The use of IV TXA has been well widely applied in total hip arthroplasty, and various studies have demonstrated its advantage in decreasing blood loss and reducing the transfusion rate [36]. Although THA is administered intravenously widely, the concerns of deep vein thrombosis or pulmonary embolism from systemic administration of TXA still exist, topical TXA administration has been proposed as an alternative option. Recently, studies have reported that topical application of TXA could reach the same hemostatic efficiency as IV administration [12].

The advantages of topical use TXA are based on that it could directly target the bleeding sites and accumulate at the surgical area, to inhibit local fibrinolytic activity, which helps stabilize fibrin clot and reduce blood loss [7]. Compared to IV TXA, topical TXA administration also possesses other advantages including easier delivery, lower systematic absorption, reduced joint swelling, and improved wound-healing [37, 38]. In addition, topical THA administration will reduce much systemic absorption the patients would develop venous thromboembolism with a much lower possibility.

However, the comparison of efficiency and safety between IV and topical TXA administration remains controversial. Some authors suggested that the topical administration of TXA could reduce blood loss and the transfusion rate, but others assumed that IV administration was a more predictable route for maximum efficacy [39]. On the other hand, other researchers found an increase in DVT, PE, and cerebrovascular strokes occur in patients who received IV TXA for hemostasis after hemiarthroplasty surgery [40]. In consideration of that aged people usually are suffering increased blood viscosity, hypertension, and arteriosclerosis, which add the risk of DVT and PE formation, these concerns need more evidence to be cleared.

Recently, some researchers have combined IV and topical use of TXA to reduce TBL in patients undergoing THA [12, 34]. Base on their results, the combined therapy significantly reduced TBL, hemoglobin drop, and blood transfusion rate [12, 34]. However, the underlying mechanism and compared safety of IV TXA, topical TXA, and the combined regimen were rarely analyzed, especially in aged patients undergoing THA with a higher risk of developing a thromboembolic complication.

We conducted a randomized controlled study in patients aged over 70 undergoing THA. The study was conducted to prove whether the combined IV TXA and topical TXA therapy could reduce blood loss and the transfusion rate, simultaneously maintain the safety regarding thrombosis in the aged population. As Table 2 showed, our results proved that 15 mg/kg of IV TXA combined with 2 g of topical TXA reach a significantly higher efficacy in reducing TBL and transfusion rates, than a single dose of IV TXA or topical TXA. Most of the blood loss from THA patients was due to the acetabular preparation, reaming cavity of the femoral canal, and incision hemorrhage, and the blood-losing process could be generally divided into an acute blood loss period (first 6 h post-surgery) and chronic blood loss period (from 6 h to 24 h post-surgery) [41]. The above study found that the peak fibrinolysis reaction level arrived 6 h after THA and then gradually decreased to the preoperative level 24 h after the operation. Thus, reducing bleeding within 24 h after surgery is a critical period for reducing TBL in THA patients. In this study, the serum antifibrinolytic activity of IV tranexamic acid can be maintained for 7 to 8 h. Therefore, IV TXA and topical TXA administration would neutralize the peak fibrinolysis reaction. As the antifibrinolytic activity of topical TXA is up to 17 h in the joint capsule, an injection of TXA in the joint capsule could continue to inhibit fibrinolysis after the peak fibrinolysis from 6 h to 24 h post-surgery. Altogether, the combined regimen worked in a coordinated way inhibited fibrin degradation and reduced blood loss caused by activation of the fibrinolysis system during the perioperative period.

The outcomes of maximum hemoglobin drop of the three groups were consistent with the TBL results. Specifically, the maximum hemoglobin drop in the combined, IV, and topical groups were 2.67 ± 0.42, 3.28 ± 0.52, and 3.75 ± 0.62 g/dL, respectively, with a significant intergroup difference (*p* < 0.01 for all). As for blood transfusion, no patient in the combined group, 6 patients in the IV group, and 7 patients in the topical group required blood transfusions. No PE was detected during the 2-month follow-up, and asymptomatic DVT was reported by 1 patient of the IV group, and 2 patients from the combined group, while the difference was not statistically significant (*p* = 0.774).

Since the antifibrinolytic mechanism of tranexamic acid is inhibition of fibrinogen activation into active fibrinogen and stopping fibrin hydrolysis [42]. So, the application of TXA does not increase fibrin formation, and thus does not promote new thrombosis.

This study still has several limitations. First, the sample size was small, and the study was only conducted in our center. A larger sample size might help find a significant difference in the prevalence of DVT or PE, which was low and not statistically different among all the groups in our study. Second, the surgical processes were not the same among patients because different patients require a different level of osteotomy and soft tissue release, which might have a specific but undefined influence on TBL and the drainage volume. Third, the current study only gave a single dose of IV TXA, and some researchers believed multiple doses of IV TXA would benefit more in reducing TBL. Fourth, the international normalized ratio (INR) or prothrombin time (PT) was not analyzed after the low molecular weight heparin was used. Different INR or PT might possess an influence on the total postoperative blood loss. Finally, in the present study, the initial conclusions in terms of the comparative safety of the three regimens need more solid evidence, due to the low incidence of DVT and PE in a limited size sample. We will start a long-term follow-up, and studies enrolled with more patients would be conducted in the future to confirm this efficient combined strategy’s safety regarding thromboembolic complications.

## Conclusion

In conclusion, the combined regimen (15 mg/kg of IV TXA and 2 g of topical TXA) significantly reduced both postoperative TBL and decreased the transfusion rate compared to a single IV dose or topical use, in aged patients over 70 undergoing primary unilateral total hip arthroplasty. This study allows us to reach initial conclusions regarding the comparative safety the combined strategy is safe even in patients aged over 70 years old, who are usually suffering a higher risk of developing thrombus.

## Data Availability

The datasets during and/or analyzed during the current study available from the corresponding author on reasonable request. All data generated or analyzed during this study are included in this published article [and its supplementary information files].

## Abbreviations

BMI: Body mass index
DVT: Deep venous thrombosis
Hb: Hemoglobin
IV: Intravenous
PE: Pulmonary embolism
TBL: Total blood loss
THA: Total hip arthroplasty
TXA: Tranexamic acid
